# Headache and musculoskeletal complaints among subjects with self reported whiplash injury. The HUNT-2 study

**DOI:** 10.1186/1471-2474-12-129

**Published:** 2011-06-08

**Authors:** Rigmor Myran, Knut Hagen, Sven Svebak, Oystein Nygaard, John-Anker Zwart

**Affiliations:** 1 Faculty of Medicine, Norwegian University of Science and Technology (NTNU), 7030 Trondheim, Norway; 2 National Centre for Diseases of the Spine, University Hospital of Trondheim, 7030 Trondheim, Norway; 3 Department of Neurology, Oslo University Hospital and University of Oslo, 0407 Oslo, Norway

## Abstract

**Background:**

To evaluate the life-time prevalence of self reported whiplash injury and the relationship to chronic musculoskeletal complaints (MSCs) and headache in a large unselected adult population.

**Methods:**

Between 1995 and 1997, all inhabitants 20 years and older in Nord-Trondelag county in Norway were invited to a comprehensive health survey. Out of 92,936 eligible for participation, a total of 59,104 individuals (63.6%) answered the question about whiplash injury (whiplash). Among these, 46,895 (79.3%) responded to the questions of musculoskeletal complaints and headache.

**Results:**

The total life-time prevalence of self reported whiplash injury was 2.9%, for women 2.7% and for men 3.0%. There was a significant association between self reported whiplash injury and headache (OR = 2.1; 95% CI 1.8-2.4), and chronic MSCs (OR = 3.3; 95% CI 2.8-3.8), evident for all ten anatomical sites investigated. The association was most pronounced for those with a combination of headache and chronic MSC for both men (OR = 4.8; 95% CI 3.6-6.2) and women (OR = 5.2; 95% CI 3.7-7.1).

**Conclusions:**

Subjects with self reported whiplash injury had significantly more headache and musculoskeletal complaints than those without, and may in part be due to selective reporting. The causal mechanism remains unclear and cannot be addressed in the present study design.

## Background

Whiplash injury occurs due to an acceleration-deceleration energy transfer to the neck resulting from motor-vehicle collisions, and the term whiplash associated disorders (WAD) was introduced in order to describe the sign and symptoms associated with the injury [1].

The prognosis of whiplash injuries show highly variable results and may be due to differences in study populations and definitions of outcome [2]. Usually the prognosis of whiplash is favorable and self-limited. The natural course for those that report symptoms after a whiplash trauma will in most cases be rapid improvement of pain and disability the first three months [3,4]. Beyond three months there is usually little improvement. It is not clear which patients are at risk of delayed recovery following whiplash injury [5,6], but a slow or poor recovery of neck pain seems to be associated with psychological factors, compensation or legal factors and initial self reported symptom severity [7]. The course of recovery in WAD is very similar to the course of neck pain in the general population [8]. Headache, neck pain and other subjective complaints are common in the general population [9-12], and both headache and neck pain are equally frequent in patients with and without a history of whiplash [13]. Headache is commonly reported after a whiplash trauma [14], but the validity of the acute and chronic whiplash headache included in the ICHD-2 criteria [15] are questionable and represents most likely occurrences of pre-accidental primary headaches like migraine and tension-type headache [16,17], The prognosis of headache after a whiplash trauma is good and similar to non-traumatized controls [18].

The construct validity of the whiplash syndrome is questionable [19], and several studies report an association between whiplash injury and a wide variety of symptoms and pain in other areas not restricted to the head and neck region [20-25]. Two studies have specifically evaluated the risk factors associated with the occurrence of wide spread bodily pain after motor vehicle collision [22,24], but these studies included insurance claimants and it is therefore not known whether there is an increased prevalence of musculoskeletal complaints (MSCs) among subject with self reported whiplash injury in the general population. Thus the main purpose of the present study was to study the relationship between self reported whiplash injury and chronic MSCs and headache in a large unselected adult population.

## Methods

### Study population

In the years 1995 to 1997, all inhabitants aged 20 years and above in the County of Nord-Trondelag of Norway were invited to participate in the Nord-Trondelag Health Study (HUNT-2). Out of 92,936 invited individuals, 66,140 (71.2%) took part in the study. The target population and the description of the participants and non-participants have been published previously [26]. In short, two questionnaires including more than 200 health-related questions were administered to the participants. The first questionnaire (Q1) was enclosed with the invitation letter and delivered when attending the health examination. The second questionnaire (Q2) was filled in after the health examination and returned by mail.

### Questionnaires

The Q1 included questions about MSCs adopted from the Standardized Nordic Questionnaire [27]. The Standardized Nordic Questionnaire has previously been evaluated and found to give reliable estimates for low-back pain, and upper limb and neck discomfort, in particular for symptoms during the past year [28,29]. In Q1 participants who responded "yes" to the question "Have you during the last year continuously for at least three months suffered from pain or stiffness in muscles and joints?" were defined as having chronic MSCs. These individuals were then asked to indicate locations by "yes" or "no" responses to the following areas of the body: neck, shoulders, elbows, wrist/hands, chest/abdomen, upper back, low back, hips, knees, and/or ankles/feet.

Subjects who answered 'yes' to the question 'Have you suffered from headache during the last 12 months?' were classified as headache sufferers. Based on data from the subsequent 12 headache questions, they were classified into two groups of either migraine or non-migrainous headache. The diagnoses were mutually exclusive. The classification of the subjects has been described in detail previously, and has been validated by interview diagnoses [30]. In short, for headache suffering the positive predictive value (PPV) was 96% and the negative predictive value (NPV) was 56%; for migraine the PPV was 84% and the NPV 78%; and for non-migrainous headache, the PPV was 68% and the NPV was 76% [30].

Whiplash injuries were investigated with the following question: "Have you ever had whiplash injury (whiplash). Out of 92,936 eligible for participation, a total of 59,104 individuals (63.6%) answered the question about whiplash injury (whiplash) in Q1. Among these, 46,895 (79.3%) responded to the questions of MSCs and headache. The subjects were classified into four groups based on the presence of headache and chronic MSC, as shown in Table 1.

**Table 1:** Demographic data

	No MSC or HA	HA-no MSC	MSC-no HA	MSC and HA
Total number	17524	8096	11195	10080
Age (mean, SD)	48.7 (17.6)	40.8 (13.7)	56.0 (15.6)	47.6 (14.0)
Gender, female %	44.3	63.3	48.6	67.1
Years of education >12 years (%)	23.7	27.4	15.4	18.3
Current smokers (%)	24.6	28.1	28.3	33.8
Analgesic use (%)*	1.4	5.3	11.7	22.1
HADS anxiety score >8 (%) HADS depression score >8 (%)	4.6 3.7	9.6 4.8	9.6 8.1	19.9 11.5
Alcohol abstainers (%)	11.0	8.2	13.3	11.7
BMI >25 (%)	57.9	52.6	66.1	60.8
High physical activity (%)**	14.6	11.7	10.3	9.6

HA = Headache, MSC = Musculoskeletal complaints

*Analagesic use daily or almost daily

**≥ 3 hours/week with hard physical activity.

### Statistical analysis

Differences between the diagnostic groups regarding demographic data (continuous and categorical) were evaluated using parametrical and non-parametric tests. P-values <0.05 were considered statistically significant. Using logistic regression, we estimated prevalence odds ratios (OR) with 95% confidence intervals (CI) for the association between self-reported whiplash injury in relation to headache and chronic MSCs. Potential confounders such as gender, age (10 years categories), duration of education (<10, 10-12 and >12 years), smoking, use of pain medication and alcohol, anxiety and depression measured by Hospital Anxiety and Depression Scale (HADS), body mass index and physical activity were adjusted for and included in the final analysis. For HADS, a cut-off value of 8 was used, as recommended in previous studies. Statistical analyses were performed using the Statistical Package for the Social Sciences (SPSS), version 15.0 (SPSS Inc., Chicago, IL, USA).

### Ethics

The Hunt-2 study was conducted in accordance with the Helsinki Declaration. The study was approved by the Regional Committee for Medical Research and Ethics in Trondheim, Norway.

## Results

The total life-time prevalence of self reported whiplash injury was 2.9%, for women 2.7%, and for men 3.0%. As demonstrated in Table 1, there was a significant difference in age between the diagnostic groups (One-way ANOVA, p < 0.0001) and for all the different categorical variables (Kruskall-Wallis test, p < 0.0001). This was most pronounced for educational level, smoking, analgesic use, physical activity, anxiety and depression among those with a combination of headache and chronic MSC. Adjustments for all these confounders were made in the final multivariate analyses. There was a significant association between whiplash injury and headache (OR = 2.1; 95% CI 1.8-2.4), and chronic MSCs (OR = 3.3; 95% CI 2.8-3.8). The association was most pronounced for those with a combination of headache and chronic MSC as shown in Table 2.

**Table 2:** Prevalence odds ratio (OR) of self reported whiplash injury in relation to diagnostic groups.

	Men			Women		
		Neck-distortion			Neck-distortion	
	Total no*	No (%)**	OR (95%CI)	Total no	%	OR (95%CI)
MSC -/HA -	9761	135 (1.4)	1.0 (ref)	7763	64 (0.8)	1.0 (ref)
MSC -/HA +	2969	66 (2.2)	1.6 (1.1-2.2)	5127	83 (1.6)	1.3 (0.9-1.9)
MSC +/HA -	5758	202 (3.5)	2.2 (1.7-2.9)	5437	134 (2.5)	2.8 (2.0-4.2)
MSC +/HA +	3315	268 (8.1)	4.8 (3.6-6.2)	6765	404 (6.0)	5.2 (3.7-7.1)

HA = Headache, MSC = Musculoskeletal complaints

Analyses adjusted for all variables in Table 1.

*Total number of subjects

**Number of subjects (and percentage) with self reported neck-distortion

The association between self reported whiplash injury and chronic MSC was evident for all ten anatomical sites for both men and women (Table 3). Among women the strongest association was found for pain in the chest and/or abdomen (OR = 7.1, 95% CI 4.9-10.4), and for both genders the association was not stronger for neck pain than upper back pain (Table 3).

**Table 3:** Prevalence odds ratio (OR) of self reported whiplash injury related to location of musculoskeletal complaints.

	Men			Women		
***Location of *MSCs**		**Neck-distortion**			**Neck-distortion**	
	Total no*	No (%)**	**OR (95% CI)**	**Total no**	**No (%)**	**OR (95% CI)**

No MSCs	12529	201 (1.6)	1.00 (ref.)	12743	147 (1.1)	1.0 (ref)
Neck	4347	384 (8.8)	5.4 (4.4-6.8)	7136	458 (6.4)	6.2 (4.9-7.9)
Shoulders	4957	326 (6.6)	3.7 (2.9-4.6)	7675	425 (5.5)	5.6 (4.4-7.2)
Elbows	1679	113 (6.7)	3.8 (2.8-5.1)	2515	140 (5.6)	5.1 (3.7-7.2)
Wrist/hands	2202	146 (6.6)	3.9 (3.0-5.1)	4577	251 (5.5)	4.9 (3.7-6.4)
Chest/abdomen	972	68 (7.0)	3.6 (2.4-5.2)	1543	107 (6.9)	7.1 (4.9-10.4)
Upper back	1421	125 (8.8)	5.0 (3.7-6.7)	3361	235 (7.0)	5.9 (4.4-7.8)
Low back	4476	255 (5.7)	3.1 (2.4-3.9)	6399	326 (5.1)	4.8 (3.8-6.3)
Hips	2693	155 (5.8)	2.9 (2.2-3.9)	5554	260 (4.7)	4.8 (3.6-6.3)
Knees	2979	170 (5.7)	3.2 (2.5-4.2)	4686	219 (4.7)	4.7 (3.5-6.3)
Ankles/feet	2179	115 (5.3)	3.0 (2.3-4.1)	3881	209 (5.4)	5.3 (3.9-7.1)

HA = Headache, MSC = Musculoskeletal complaints. Analyses adjusted for all variables in Table 1.

*Total number of subjects with localized MSCs complaints in the population.

**Number of subjects (and percentage) with self reported neck-distortion

Among those with chronic MSC, 39.3% of the subjects with whiplash injury reported that these complaints had a significant impact on their work ability compared to 25.5% of those with chronic MSC but without self reported whiplash injury (p < 0.0001). There was also a significant difference in impact on leisure activities between these two groups (75.4% versus 61.3%, p < 0.0001). The association between self reported whiplash injury and headache was found for both migraine (OR = 2.2; 95% CI 1.8-2.6) and non-migrainous headache (OR = 2.1; CI 1.7-2.4) for both genders (data not shown).

In short, for headache suffering the positive predictive value (PPV) was 96% and the negative predictive value (NPV) was 56%; for migraine the PPV was 84% and the NPV 78%; and for non-migrainous headache, the PPV was 68% and the NPV was 76% [30].

## Discussion

In this large population-based, cross-sectional study, self-reported whiplash injury was associated with increased prevalence of headache and chronic MSCs, which was evident for all anatomical sites. Individuals with a combination of headache and chronic MSC were five times more likely to report whiplash injury than those without any complaints.

The life-time prevalence of having sustained a whiplash trauma in our study was 2.9% and is much lower than the 15.9% reported in a population-based cross-sectional study among the Saskatchewan population [20]. Published reports from the Confederation of Norwegian Enterprise comprising all the Norwegian government insurance companies indicate that the most common cause of whiplash, a rear end collision, in year 2000 involved 54000 vehicles and half of these were hit from behind, i.e. 27000 people. Over a 10 year period one can calculate that approximately 270 000, i.e. 6% of the population, will be involved in such collision. In addition comes all other traffic- and sport accidents. This means that the percentage who have sustained on an accident with whiplash mechanism within a relevant time period is probably much higher than the 2.9% who self report a whiplash accident [31]. It is therefore very likely that whiplash traumas were grossly underreported in the present study, and that selective reporting, e.g. among those with complaints may be present.

The present results are in accordance with previous cross-sectional studies, reporting a wide variety of health complaints among persons with self-reported whiplash injury [20,21] and a Swedish cohort study showed that persons with chronic pain after a whiplash injury had an increased risk for pain from different anatomical sites [32]. There are some that argue that these symptoms might reflect central sensitization [33] but there is also a strong association between whiplash injury and psychiatric disorders [23], which might reflect a reversed causality, that is, increased risk of future self-reported whiplash injury in individuals who already have anxiety and depression [34]. Thus, the strong relationship between whiplash injury and the combination of headache and chronic MSC in the present study may, at least in part, reflect personality traits rather than biological mechanisms [35]. It should be emphasized that in studies dealing with subjective complaints like headache, musculoskeletal pain and psychiatric symptoms, the results may be influenced by a tendency to answer in a similar way all questions regarding complaints ("reporting bias") [36]. The results from the present cross-sectional study must be evaluated with caution. It cannot be determined whether whiplash injury causes neither MSCs nor headache, or whether other risk factors or a shared susceptibility causes these associations. Secondly, since both headache, chronic MSCs and whiplash injury are based on self-report, individuals with neck pain and other pain are more likely to remember and report a previous neck trauma than those without complaints, i.e. differential information (recall bias) [37].

Even though the use of validated questionnaires reduces the risk for misclassification, the questionnaire-based diagnoses are not optimal when compared to interview diagnoses. There is a possibility of non-differential misclassification of diagnosis that might weaken real associations, but we think this is a minor problem as the prevalence of headache and MSC in the current population is consistent with data from other population-based studies in the Western countries [10,12,38]. In addition the large and unselected population and the high participating rate, reduces the risk of selection bias. Selective participation was unlikely, since neither headache, neck-distortion nor chronic MSCs were the main objectives. The impact of non-participants has been discussed in more detail previously [26], but the large sample size decreased the risk of chance findings and the wide range of data made it possible to adjust for potential confounding variables.

## Conclusions

Subjects with self reported whiplash injury had significantly more headache and musculoskeletal complaints than those without, and may in part be due to selective reporting. The causal mechanism remains unclear and cannot be addressed in the present study design.

## Competing interests

The authors declare that they have no competing interests.

## Authors' contributions

RM conceived of the study and performed the statistical analysis. RM drafted the manuscript with input from the other authors. All authors read, revised and approved the final manuscript.

## Pre-publication history

The pre-publication history for this paper can be accessed here:

http://www.biomedcentral.com/1471-2474/12/129/prepub
